# 

**Published:** 1877-03-20

**Authors:** 


☞ All communications relating to the business of THE RECORD, for the year 1877, must be addressed to
DR. R. C. WORD,
Business Manager, South. Med. Rec.,
Atlanta, Ga.

☞ Brief and practical communications are solicited on all subjects pertaining to medicine, also reports of cases in practice.

☞ Send money by check, postal order, or registered letter.

☞ Write your name, post-office, county and State plainly.

☞ Quite a number of our readers who have received the January and February numbers of the RECORD, have not yet remitted the amount of subscription. Our friends will please remember that we are now on the cash basis.

☞ See advertisement of A. R. Everett, manufacturing Jeweler and Engraver. Mr. Everett is an affable and pleasant gentleman, highly skilled in his employment and merits the patronage of the public.

☞ Two or three parties have returned copies sent them, thereby declining to subscribe, but, not sending us their names we cannot know who they are.

☞ The Improved Trommer's Extract of Malt is likely to prove a very useful and popular agent with the profession. It is advertised in the present issue.

### COLABORATORS AND CONTRIBUTORS

L. B. Anderson, M.D., Va.
Swan M. Burnett, M.D., D. C.
J. R. Bristow M.D., Texas.
J. W. Booth, M.D., N. C.
W. F. Barr, M.D., Va.
B. W. Corn, M.D., Texas.
John Castor, M.D., Iowa.
E. T. Easley. A.M., M.D., Ark.
A. H. Goelet, M.D., N. Y.
E. C. Gehrung, M.D., Mo.
G. D. Hodge, M.D., Ark.
S. M. Hogan, M.D., Ala.
A. McLane Hamilton, M.D.,N.Y.
Z. B. Herndon, M.D., Va.
Lindsay Johnson, M.D., Ga.
A. R. Kilpatrick, M.D., Tex.
R. Kells, M.D., Miss.
W. L. Lipscomb, M.D,, Miss.
J. M. Lewis, M.D., Miss.
C. H Lathrop, M.D., Iowa.
A. A. Lyon, M.D., La.
M. Lewis, M.D., Tenn.
G. M. Rivers, M.D., S. C.
A. S. Payne, M.D., Va.
A. M. Whitehead, M.D., O.

